# Temporal hampering of thyroid hormone synthesis just before hatching impeded the filial imprinting in domestic chicks

**DOI:** 10.3389/fphys.2023.1084816

**Published:** 2023-02-16

**Authors:** Shouta Serizawa, Naoya Aoki, Chihiro Mori, Toshiyuki Fujita, Shinji Yamaguchi, Toshiya Matsushima, Koichi J. Homma

**Affiliations:** ^1^ Department of Molecular Biology, Faculty of Pharmaceutical Sciences, Teikyo University, Itabashi-ku, Tokyo, Japan; ^2^ Department of Biological Sciences, Faculty of Pharmaceutical Sciences, Teikyo University, Itabashi-ku, Tokyo, Japan; ^3^ Department of Biology, Faculty of Science, Hokkaido University, Hokkaido, Japan

**Keywords:** filial imprinting, thyroid hormone, methimazole (MMI), domestic chicks (gallus gallus), development, learning process

## Abstract

Thyroid hormones play a critical role in the initiation of the sensitive period of filial imprinting. The amount of thyroid hormones in the brains of chicks increases intrinsically during the late embryonic stages and peaks immediately before hatching. After hatching, a rapid imprinting-dependent inflow of circulating thyroid hormones into the brain occurs *via* vascular endothelial cells during imprinting training. In our previous study, inhibition of hormonal inflow impeded imprinting, indicating that the learning-dependent inflow of thyroid hormones after hatching is critical for the acquisition of imprinting. However, it remained unclear whether the intrinsic thyroid hormone level just before hatching affects imprinting. Here, we examined the effect of temporal thyroid hormone decrease on embryonic day 20 on approach behavior during imprinting training and preference for the imprinting object. To this end, methimazole (MMI; a thyroid hormone biosynthesis inhibitor) was administered to the embryos once a day on days 18–20. Serum thyroxine (T_4_) was measured to evaluate the effect of MMI. In the MMI-administered embryos, the T_4_ concentration was transiently reduced on embryonic day 20 but recovered to the control level on post-hatch day 0. At the beginning of imprinting training on post-hatch day 1, control chicks approached the imprinting object only when the object was moving. In the late phase of training, control chicks subsequently approached towards the static imprinting object. On the other hand, in the MMI-administered chicks, the approach behavior decreased during the repeated trials in the training, and the behavioral responses to the imprinting object were significantly lower than those of control chicks. This indicates that their persistent responses to the imprinting object were impeded by a temporal thyroid hormone decrease just before hatching. Consequently, the preference scores of MMI-administered chicks were significantly lower than those of control chicks. Furthermore, the preference score on the test was significantly correlated with the behavioral responses to the static imprinting object in the training. These results indicate that the intrinsic thyroid hormone level immediately before hatching is crucial for the learning process of imprinting.

## 1 Introduction

Newly-hatched chicks undergo filial imprinting, in which they follow and memorize their mother in order to receive sufficient care (Lorenz, 1937). The filial imprinting of domestic chicks is a useful model for early learning (Horn, 1986; Matsushima et al., 2003; McCabe, 2019). Newly hatched chicks tend to innately follow a conspicuous moving object (Bateson, 1966; Bolhuis, 1991; McCabe, 2013; Vallortigara and Versace, 2018). After repeatedly following an object, they prefer it (Bateson, 1964; Bateson, 1966). Imprinting has a sensitive period from approximately days 1–3 in domestic chicks (Ramsay and Hess, 1954; Jaynes, 1957; Yamaguchi et al., 2012). The neuronal and molecular mechanisms of imprinting acquisition and the sensitive period have been investigated extensively in chicks that are imprinted in a laboratory setting (Horn, 1998, 2004; Maekawa et al., 2006; Yamaguchi et al., 2012; Solomonia and McCabe, 2015; Aoki et al., 2018; Aoki et al., 2020; Aoki et al., 2022b; Mori et al., 2022). Triiodothyronine (T_3_), a thyroid hormone, plays a critical role in imprinting acquisition at the start of the sensitive period (Yamaguchi et al., 2012). T_3_ levels in the brain begin to increase from embryonic day 15 (E15) and then peak immediately before hatching. T_3_ levels also increase during imprinting training after hatching through the inflow of circulating thyroid hormones into the brain. Inhibition of inflow prevents imprinting acquisition (Yamaguchi et al., 2012). These data indicate that the rapid inflow of circulating thyroid hormones into the brain *via* vascular endothelial cells initiates the sensitive period of imprinting.

Thyroid hormones also play an important role in vertebrate brain development. They induce neuronal migration, proliferation, neurite outgrowth, synaptogenesis, and myelination during embryonic development (Bernal, 2007; Darras et al., 2009). These indicate that thyroid hormones during the prenatal period are necessary for normal neurogenesis, the structural basis of cognitive functions. In rats, prenatal hypothyroidism induced by exposing pregnant mothers to antithyroid drugs impairs the learning of their offspring, such as passive avoidance, alternative discrimination, and spatial tasks (Akaike et al., 1991; MacNabb et al., 1999; Darbra et al., 2004; Gilbert and Sui, 2006; Shafiee et al., 2016). In domestic chicks, prenatal exposure to methimazole (MMI; a thyroid hormone biosynthesis inhibitor) at E14 impaired the discrimination between imprinting and control objects and approach behavior toward objects (Kagami et al., 2010; Haba et al., 2014). In these studies, a single MMI administration at E14 prolonged the embryonic duration from E21 to E24 in some cases, delaying the hatching date by 3 days. These data suggest that thyroid hormones decrease in the middle of the embryo stages delays ontogeny and thus impairs imprinting.

By the end of the embryonic stages, brain development mostly matures to execute cognitive functions after hatching. During the late embryonic stages, the gene expression of type 2 iodothyronine deiodinase, which catalyzes the conversion of T_4_ to T_3_, increases dynamically with brain development from approximately E18, and brain thyroid hormone (T_3_) levels reach a peak at E20, suggesting that increased T_3_ plays an important role in cognitive brain functions such as imprinting (Reyns et al., 2003; Darras et al., 2009; Yamaguchi et al., 2012; Van Herck et al., 2013; Ahmed, 2015; Too et al., 2017). However, it remains unknown whether thyroid hormone in the late embryonic stage such as the pre-hatching period of E20 is necessary for imprinting. The thyroid hormone in the late embryonic stages may be involved in the maturation of neural circuits underlying the basis for filial imprinting. We assumed that imprinting might be impaired without severe damage to the ontogeny if thyroid hormone synthesis is temporally suppressed by MMI administration at late stages of development, just before hatching.

We recently developed a running disc-based behavioral apparatus to analyze the imprinting learning process (Aoki et al., 2022a). This apparatus records the approach behavior toward the imprinting object in milliseconds. With the experimental behavioral procedure we designed, this apparatus was able to evaluate the three learning processes involved in imprinting. The first is the “spontaneous approach behavior” toward the moving imprinting object. The second is the “acquired approach behavior” toward the static state of the imprinting object. The third is “discrimination following approach behavior” between imprinting and control objects. During the training, the approach distances to the moving object were significantly correlated with those of the static object. In addition, the approach distances to the static object in training were significantly correlated with preference scores in the test. This indicates that the chicks proceeded stepwise from the first to the third imprinting learning process. At 30 h post-hatching, the chicks reached the third learning process, but the chicks before that age remained in the first or second learning processes (Aoki et al., 2022a). We think that this apparatus is useful for evaluating the effects of temporal thyroid hormone decrease caused by exposure to MMI during the pre-hatch period on the learning processes in imprinting after hatching.

This study aimed to examine the effect of temporal thyroid hormone decrease by MMI administration during the pre-hatch period of E20 on the learning processes and preference for imprinting objects at P1. To achieve this, we injected MMI into the eggs’ air chamber from E18 to E20 and then conducted imprinting training and tests with a running disc-based behavioral apparatus at P1. In addition, we measured serum levels of thyroxine (T_4_). The results revealed that a sufficient thyroid hormone concentration at pre-hatch periods from E18 to E20 is crucial for imprinting, especially for the learning processes, i.e., both “spontaneous approach behavior” toward the moving imprinting object and “acquired approach behavior” toward the static imprinting object.

## 2 Materials and methods

### 2.1 Animals

The experiments were conducted following the guidelines of the National Regulations for Animal Welfare in Japan, with the approval of the Committee on Animal Experiments of Teikyo University (approval number:18-015). Domestic chicks (*Gallus domesticus,* Cobb strain) were used in this study. Fertilized eggs were obtained from a local supplier (3-M; Aichi, Japan) and incubated at 37°C for 21 days. One hundred and fifty eggs were incubated. Because 18 chicks in the MMI-administered group and 10 chicks in the PBS-administered group hatched before the third administration, we did not use them in this study. In total, one hundred and seven chicks hatched after the third administration on P0. Of them, fifty-three were used in this study. The remaining 54 chicks were used for the other research. Twenty-one chicks were used for the behavioral experiments. Thirty-two chicks were used for the measurement of the concentrations of T_4_ in the serum. The chicks were hatched in an incubator in the dark. Within 1 h of hatching, the chicks were placed in dark plastic enclosures in a breeder at 32°C to prevent light exposure until behavioral experiments (Izawa et al., 2001). After the experiment, the chicks were sexed using PCR. Sex ratios were approximately 50% in both the behavioral experiment and the experiment measuring the concentrations of T_4_ in the serum. We merged the data of both sexes since no sex differences appeared at the population level. After behavioral experiments or blood sampling, the birds were euthanized with an overdose of isoflurane.

### 2.2 MMI injection into the fertilized eggs

**FIGURE 1:**
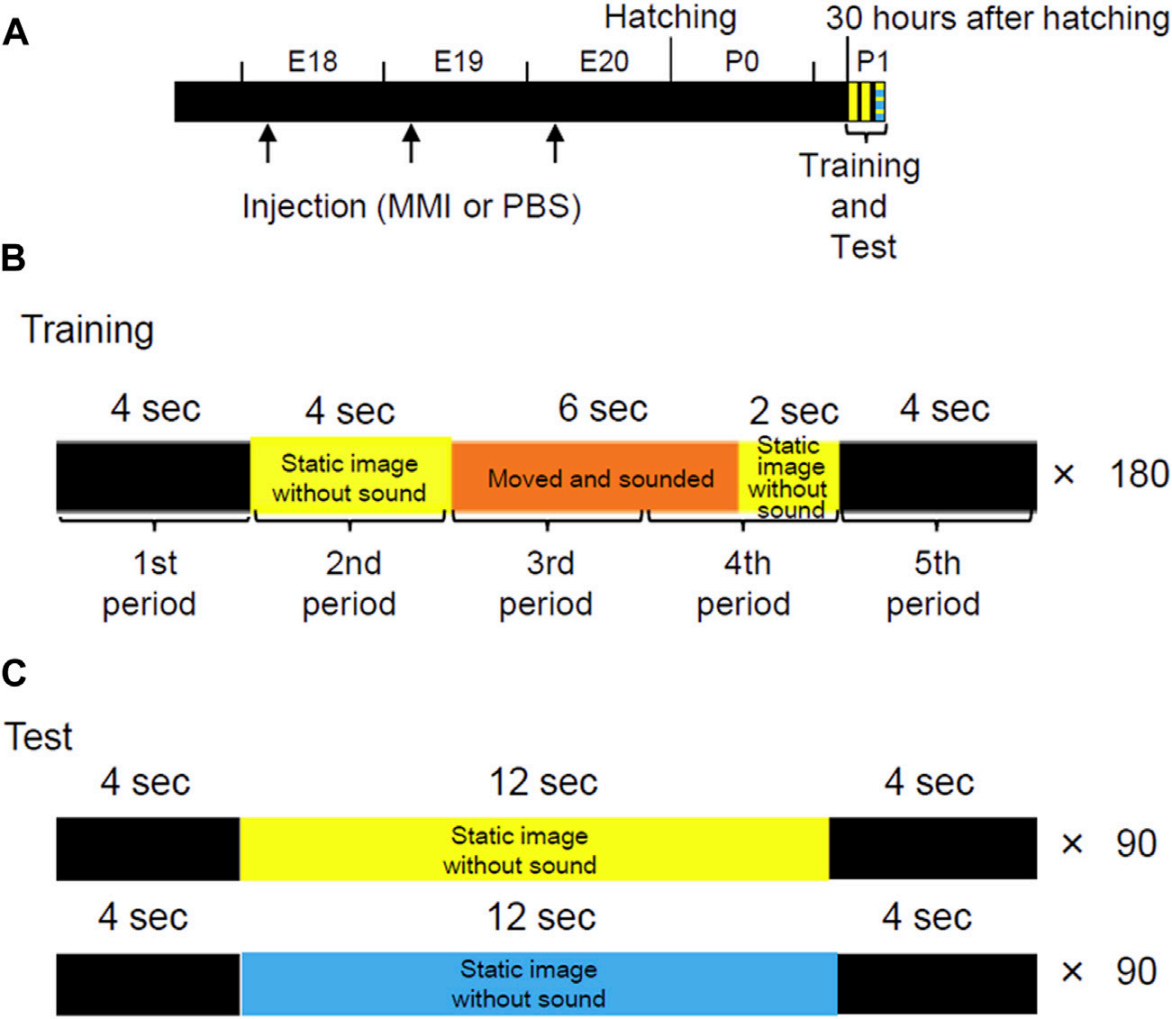
Experimental procedures to observe the effects on imprinting of thyroid hormone synthesis decrease in the pre-hatch period. **(A)** Schematic representation of the experimental procedure. The incubated eggs were injected with methimazole (MMI) or phosphate-buffered saline (PBS) once per day from embryonic day (E) 18 to E20. Imprinting training and tests were conducted 30 h after hatching. These methods are similar to those in the study performed by Aoki et al., 2022a. **(B)** In one session of training, a movie, 20 s long, was repeatedly played on the monitor 180 times for 1 h. In the training movie, a yellow imprinting object appeared for 12 s. The object was static for the first 4 s, then repeatedly turned clockwise and anti-clockwise with artificial sounds for 6 s. After the object stopped moving, it remained static again for the last 2 s. A trial of 20 s was divided into five periods (4 s each) to analyze the performance of the chicks during training. **(C)** In the preference test, two types of movies (20 s each) were played pseudo-randomly for 1 h. In one movie, the yellow imprinting object appeared for 12 s and remained static without making a sound. In another movie, a blue control object appeared for 12 s and remained static without sound.

The incubated eggs received a single MMI injection per day from E18 to E20 (Figure 1A). Two hundred microliters of MMI solution (10 μmol/egg) were injected into the air chamber through a hole on the round edge of the shell. Plastic paraffin film (Parafilm, Bemis Company Inc., Neenah, United States) was used to seal the holes. MMI, the thyroid hormone biosynthesis inhibitor, was dissolved in the phosphate-buffered saline (PBS). For the control, the other incubated eggs received injections of PBS solution at the same volume and schedule.

### 2.3 Disc-based apparatus for imprinting training

We used the running disc-based behavioral apparatus (25 cm in diameter) used in a previous study (Aoki et al., 2022a). The imprinting object was displayed on a thin-film transistor monitor (refresh rate: 56–75 Hz; size: 213 mm × 160 mm; type: LCM-T102AS, Logitec Corp., Tokyo, Japan). When the chick approached the imprinting object on the monitor, the disc rotated clockwise. Therefore, after the chick approached the monitor, the chick was brought back to its opposite side by his/her weight because the slope of the disc was slightly steep. This apparatus sent a transistor-transistor logic signal at every 1/10 turn of the disc to a personal computer through an OmniPlex data acquisition system (Plexon Inc., Dallas, United States). The sampling frequency of the recording was 2,000 samples/s. The approach distance was calculated from the recorded data of the transistor-transistor-logic signals. Because the estimated approach distance in one lap was approximately 40 cm, one transistor-transistor-logic signal was a 4.0 cm approach toward the monitor by the chick. The approach distance was the distance that a chick approached the object during the training and the test.

### 2.4 Imprinting training using disc-based apparatus

The imprinting training was performed as described by Aoki et al. (2022a), and briefly outlined as follows: Two sessions (Training one and 2) were conducted at 1-h intervals at P1 and 30 h after hatching (Figure 1A). One training session involved playing a movie (20 s long) 180 times for 1 h (Figure 1B). The movie was created using the 3D creation software Blender (Blender Foundation, Amsterdam, Netherlands). In the training movie, a yellow, imprinting object appeared for 12 s. The object was static for the first 4 s and then repeatedly turned clockwise and anti-clockwise with artificial sounds from two speakers for 6 s. After the object stopped moving and making sounds, the static object appeared again for the last 2 s. The interval between the appearances of the object was 8 s. During this interval, a black screen appeared on the monitor. A trial of 20 s was divided into five periods (4 s per period) to analyze the performance of the chicks during training. Subsequently, the approach distance for each period was calculated.

### 2.5 Preference test using disc-based apparatus

The preference test was conducted 1 h after Training 2. For the preference test, two types of movies (20 s each) were played pseudo-randomly for 1 h using the disc apparatus (Figure 1C). In one movie, the yellow, imprinting (familiar) object appeared for 12 s and did not move or make a sound. During the remaining 8 s, the monitor presented a black screen. In the other movie, a blue control (unfamiliar) object appeared for 12 s and did not move or make a sound. In the previous study, we showed chicks do not have innate preferences for either yellow or blue objects (Aoki et al., 2022a). The approach distance during the appearance of the object (12 s) was measured and averaged for each trial. The preference score was calculated by “approach distance to the imprinting object/(approach distance to the imprinting object + approach distance to the control object).”

### 2.6 Measurement of T_4_ in the serum by ELISA analysis

We measured the serum concentration of circulating T_4_ at several developmental stages in chicks to examine the effects of MMI injection. Blood was sampled by puncturing the medial metatarsal vein in the lower leg 1, 12, or 24 h after hatching (PBS, *n* = 10; MMI, *n* = 9). At E20, blood was sampled by cutting the blood vessels of the chorioallantoic membrane 12 h before hatching when embryos were externally pipped (PBS: *n* = 7; MMI: *n* = 6). Blood serum was isolated by centrifuging whole blood at 5,000 × *g* for 25 min at room temperature (approximately 24°C) and was stored at −30°C until T_4_ analysis. Serum T_4_ levels were measured using an immunoassay kit (Abor Assays, Ann Arbor, MI, United States; Cat. No. K050-H1) following the manufacturer’s protocol (Mishra et al., 2017; Prabhat et al., 2020). Briefly, 5 µL of serum samples were incubated with dissociation reagent at a 1:1 dilution for 60 min at room temperature (approximately 25°C). Next, assay buffer was added to obtain a sample volume of 850 μL at a final dilution of 1:170. Next, we pipetted 100 µL of each standard and diluted samples to designated wells, as well as 125 μL and 100 µL of assay buffer to the non-specific binding and maximum binding wells, respectively. Then, 25 µL each of T_4_ conjugate and antibody was added (except for the non-specific binding wells), and the plate was incubated at room temperature (approximately 25°C) on a plate shaker at 700 rpm for 1 h. After washing the plate four times with a wash buffer, 100 µL of TMB (3,3′,5,5′-tetramethylbenzidine) substrate was added to each well, and the plate was incubated for another 30 min without shaking at room temperature (approximately 25°C). After adding 50 µL of stop solution to each well, the plate was read at 450 nm using an iMark microplate reader (Bio-Rad Laboratories, Hercules, United States).

### 2.7 Statistical analyses for behavioral data

R software (R Development Core Team) or MATLAB (Mathworks, Natick, MA, United States) for Windows was used for statistical analyses. The numbers of animals used are shown in the figures. The equalities of the variance of the behavioral data and T_4_ levels were checked using an *F*-test. Because the results of the *F*-test showed that the variances were not equal, Welch’s *t*-test was used as a non-parametric test. Welch’s *t*-test was used to analyze both the behavioral data and T_4_ levels and compare the data between MMI- and PBS-administered chicks. The *p* values of the tests are presented in Supplementary Table S1. We also conducted Welch’s *t*-test to estimate the differences between the approach distances to the yellow object and those to the blue object during the test for individual chicks. Differences were considered statistically significant at *p* < 0.05. Pearson’s product-moment correlation was applied to test the significance of the correlation between the preference score and behavioral data in training. We constructed generalized linear mixed models (GLMM) in MATLAB and evaluated them using the Akaike information criterion (AIC). We adopted the probit function as a link. In both Pearson’s product-moment correlation and the GLMM, data obtained from the PBS- and MMI-administered chicks are included.

## 3 Results

### 3.1 Effects of the MMI injection on hatching date, hatching rate, and body weight

The hatching dates of MMI-administered chicks were not different from those of PBS-administered chicks, showing that MMI administration from E18 to E20 did not delay the hatching date. The hatching rate in the PBS-administered group was 90.0% (Table 1). On the other hand, the hatching rate of the MMI-administered group was approximately 5% lower than that of the PBS-administered group. In addition, the body weight of the MMI-administered group was not significantly different from that of the PBS-administered group. These data indicate that the MMI administration did not cause severe deficits in the development of chicks.

**TABLE 1:** Hatching rate and body weight.

	Hatching rate (%)	Body weight (g)
PBS	90.0 (54/60)	40.5 ± 0.5
MMI	85.5 (53/62)	41.3 ± 0.9

Mean ± SEM.

### 3.2 Effects of MMI injection on the concentration of T_4_ in the serum

**FIGURE 2:**
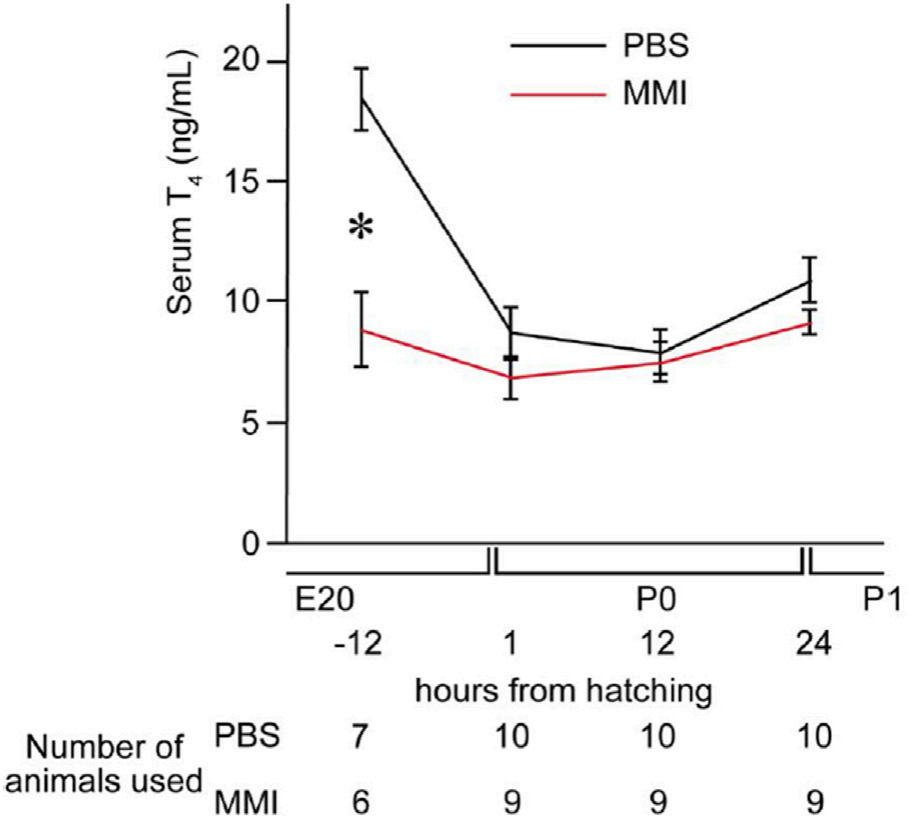
Effects of methimazole (MMI) injection on the concentrations of T_4_ in the serum. We measured T_4_ concentration in the serum 12 h before hatching (external pipping) and at 1, 12, and 24 h after hatching. At 12 h before hatching, T_4_ concentrations in the serum of MMI-administered chicks were significantly lower than those of PBS-administered chicks. However, the T_4_ concentrations did not differ between the two groups at the other three time points. Data are presented as the mean ± SEM. Asterisks (*) indicate significance (*p* < 0.05).

Serum T_4_ concentrations were measured 12 h before hatching and at 1, 12, and 24 h after hatching (Figure 2) to examine the effect of the MMI injection. At 12 h before hatching, the concentrations of T_4_ in the serum of MMI-administered chicks were significantly lower than those of PBS-administered chicks. Concentrations did not differ between the two groups from 1 to 24 h after hatching. This result indicates that the concentrations of T_4_ were lowered by MMI administration 12 h before hatching, and the concentrations of T_4_ had already recovered at the start of the imprinting training 30 h after hatching at P1. The data suggest that the artificial thyroid hormone decrease was transient during the pre-hatch period and that imprinting training and the test were executed at the regular hormonal level, similar to the control chicks.

### 3.3 Effects of MMI injection on the behavior during the imprinting training

**FIGURE 3:**
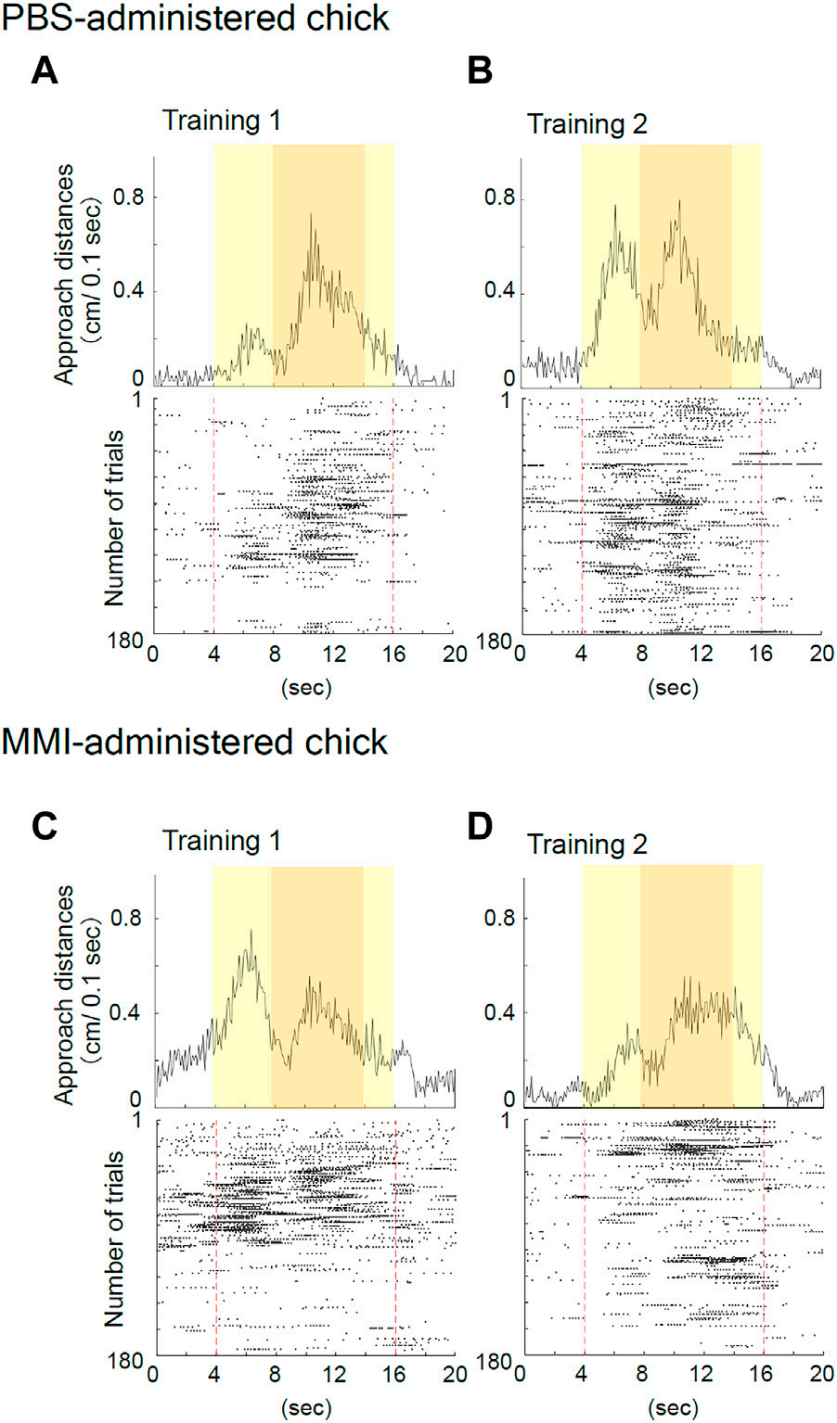
Representative behavior of phosphate-buffered saline (PBS)-administered and methimazole (MMI)-administered chicks during imprinting training. **(A)** Averaged approach distances (upper column) and raster plots (lower column) for each trial in Training one of the PBS-administered chick are shown. In the raster plots, one plot indicates the timing of a 1/10 turn of the disc, and the broken red line indicates the onset and offset of the object presentation. The PBS-administered chick responded to the object when it was moving and made a sound during Training 1. **(B)** The PBS-administered chick responded to both the moving and static imprinting object during Training 2. **(C)** The MMI-administered chick responded to the imprinting object during Training one similar to the PBS-administered chicks. **(D)** The MMI-administered chick’s responses decreased for the imprinting object during Training 2.

The representative behavior of PBS-administered chicks during training is shown in Figures 3A,B. During the initial part of Training 1, the PBS-administered chick responded to the object only when it was moving and made a sound (Figure 3A). This “spontaneous approach behavior” toward the moving object has been defined as the first learning process in imprinting (Aoki et al., 2022a). After repeatedly approaching the moving object, the chick became to approach the object, also when it was not moving (Figures 3A,B). This “acquired approach behavior” has been defined as the second learning process in imprinting. The data showed reproducibility in different animal group settings used in the study by Aoki et al.

The representative behavior of MMI-administered chicks during training is shown in Figures 3C,D. The MMI-administered chick during Training one approached both the moving and static state of the object (Figure 3C). However, during Training 2, the chick’s responses decreased for the imprinting object (Figure 3D).

**FIGURE 4:**
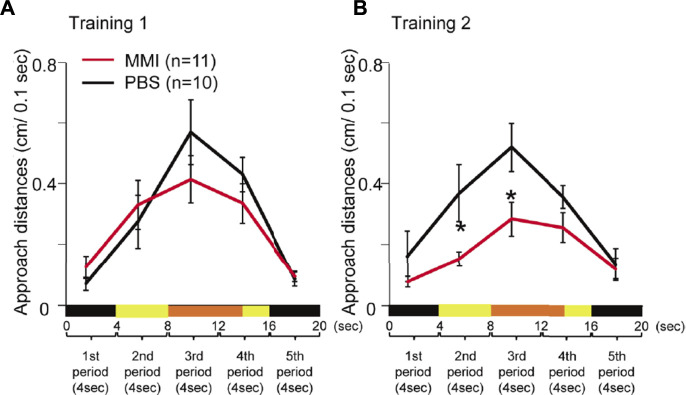
The behavior of phosphate-buffered saline (PBS)-administered and methimazole (MMI)-administered chicks during imprinting training. **(A)** Averaged approach distances during Training one are shown. A trial of 20 s was divided into five periods (4 s each). The black line indicates the approach distances in PBS-administered chicks. The red line indicates the approach distances of MMI-administered chicks. The approach distances of MMI-administered chicks were not significantly different from those of PBS-administered chicks. **(B)** During Training 2, the approach distances of MMI-administered chicks in the second and third periods were significantly shorter than those of PBS-administered chicks. Data represent mean ± SEM. The asterisks (*) indicate significance (*p* < 0.05).

The average behavioral data are presented in Figure 4. During Training 1, MMI-administered chicks approached the monitor from the second period to the fourth period when the imprinting object appeared (Figure 4A). The approach distances of the MMI-administered chicks during these periods were not significantly different from those of PBS-administered chicks. However, during Training 2, the approach distances of the MMI-administered chicks in the second and third periods were significantly shorter than those of the PBS-administered chicks (Figure 4B). This shows that they approached similarly to the PBS-administered chicks during Training 1, but the responses to the object decreased during Training 2. This result indicates that the MMI-administered chicks acquired approach behavior the same as the PBS-administered chicks during Training 1, but could not keep the approach behavior during Training 2.

### 3.4 Effects of MMI injection on the preference score

**FIGURE 5:**
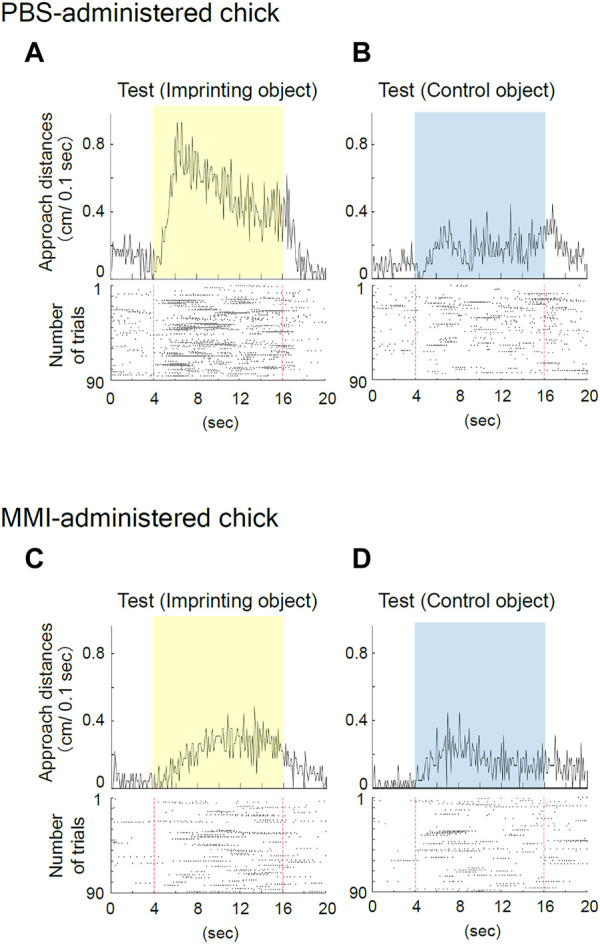
Representative behavior of phosphate-buffered saline (PBS)-administered and methimazole (MMI)-administered chicks during imprinting test. **(A)** Averaged approach distance and raster plots for the imprinting object trials in the test of the PBS-administered chick are shown. The chick constantly responded to the static state of the object. **(B)** The PBS-administered chick responded to the control object, but the responses were small. **(C)** The MMI-administered chicks responded weakly to the imprinting object. **(D)** The MMI-administered chick responded slightly to the control object as they did to the imprinting object.

The representative behavior of PBS-administered chicks during the preference test is shown in Figures 5A,B. When the yellow, imprinting object was presented, the PBS-administered chicks constantly responded to the static state of the object, as shown in the raster plots (Figure 5A). When the blue control object was presented, the chicks weakly responded to the object (Figure 5B). Thus, the preference score is high (0.74). This result again shows experimental reproducibility in the third learning process, the status of the “discrimination following approach behavior” between the two objects in the test (Aoki et al., 2022a).

The representative behavior of MMI-administered chicks during the preference test is shown in Figures 5C,D. Regarding MMI-administered chicks, the responses to both the yellow and blue controls were smaller and showed similar properties (Figures 5C,D). In this case, the preference score was low (0.58), indicating poor preference. This indicates that MMI administration caused severe functional damage to imprinting memory.

**FIGURE 6:**
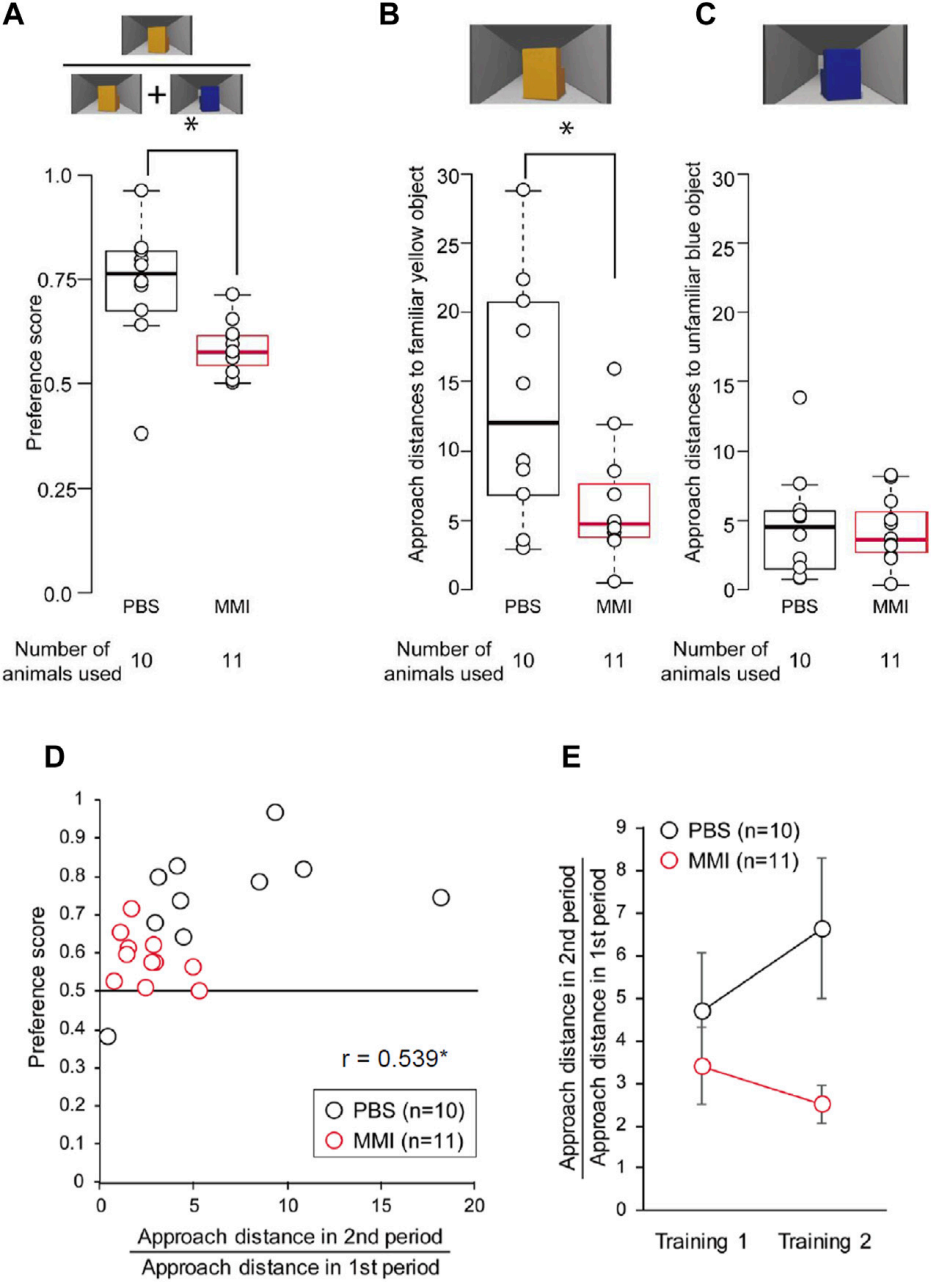
The behavior of phosphate-buffered saline (PBS)-administered and methimazole (MMI)-administered chicks during imprinting test. **(A)** Preference scores of the PBS-administered and the MMI-administered chicks in the imprinting test are shown. Preference scores of the MMI-administered chicks were significantly less than those of the PBS-administered chicks. **(B)** Approach distances to the familiar yellow object are shown. The approach distances to the imprinting object of the MMI-administered chicks were significantly less than those of the PBS-administered chicks. **(C)** The approach distances to the unfamiliar blue object are shown. The approach distances to the control object of the MMI-administered chicks were not significantly different from those of the PBS-administered chicks. **(D)** The scatter plots between the preference score and approach distance in the second period are normalized by that in the first period. Black dots indicate PBS-administered chicks. Red dots indicate MMI-administered chicks. **(E)** The normalized approach distance in the second period in the Training one and Training two are shown. Data represent mean ± SEM. The asterisks (*) indicate significance (*p* < 0.05).

The average preference scores are shown in Figure 6A. The preference scores of MMI-administered chicks were significantly lower than those of PBS-administered chicks. In the PBS-administered chicks, nine of ten chicks showed significant differences between approach distances to the yellow object and those to the blue object during the test. On the other hand, in the MMI-administered chicks, two of eleven chicks showed significant differences (Supplementary Table S2), indicating that most of the MMI-administered chicks were unable to reach the third learning process, “discrimination following approach behavior.”

We analyzed the chicks’ behavior during the preference test in more detail. The approach distances to the familiar yellow object of the MMI-administered chicks were significantly shorter than those of the PBS-administered chicks (Figure 6B). In addition, the approach distances to the unfamiliar blue object were relatively short in both groups and did not differ between the two groups (Figure 6C). These results suggest that the MMI-administered chicks responded less to the imprinting object than the PBS-administered chicks. The chicks did not have a sufficient preference for the object to discriminate it from the unfamiliar object.

Lastly, we plotted the preference score against the approach distance in the second period normalized by the distance in the first period in Training two to examine whether the preference score is associated with approach distances during Training 2. When the Pearson’s product-moment correlation was applied to the data including both the PBS-administered chicks and MMI administered chicks, the correlation between the preference score and normalized approach distance in the second period was significant (*r* = 0.539, *p* < 0.05, Figure 6D). This normalized approach distance in the second period of Training two increased in PBS-administered chicks compared to that in Training 1 (Figure 6E). However, the approach distance decreased in the MMI-administered chicks. This indicates that the MMI prevented the chicks from approaching the static object, causing impairment of the preference for the imprinting object. Further, a statistical analysis using generalized linear mixed models (GLMM) revealed that the approach distance in the second period was a more appropriate element for acquiring a higher preference for the imprinting object than in other periods (see Supplementary Statistics for details). These indicate that the thyroid hormone just before hatching may be involved in the development of the mechanisms for the facilitation or maintenance of the acquired approach behaviors.

## 4 Discussion

In this study, we showed that MMI administration from E18 to E20 impaired the learning process of “spontaneous approach behavior” and “acquired approach behavior” (Training 2). In addition, MMI-administered chicks did not discriminate between the imprinting object and the control object (preference test). We also showed that the circulating T_4_ in MMI-administered chicks at E20 was significantly lower than those in the control chicks. However, the concentrations recovered to the control levels at the hatching of P0. The MMI administration did not affect the hatching date, hatching rate, and body weight. These data suggest that MMI administration temporarily suppressed the concentration of thyroid hormones during the pre-hatching period but did not delay development. The results revealed that a sufficient thyroid hormone concentration at pre-hatch periods from E18 to E20 is crucial for imprinting, especially for “spontaneous approach behavior” and “acquired approach behavior”. We assume that thyroid hormones play a critical role in brain development during the pre-hatching period, which is necessary for imprinting after hatching. Neurogenesis in the brain is mostly completed before hatching in precocial birds. However, the synaptic maturation of neural circuits underlying the basis for cognitive functions will continue during the late pre-hatch period, even after hatching. If that is the case, the defects caused by the lack of sufficient thyroid hormones just before hatching might have caused the impairment of the cognitive function of imprinting.

Thyroid hormones are involved in brain development, including neuronal migration, proliferation, neurite outgrowth, synaptogenesis, and myelination (Bernal, 2007; Darras et al., 2009). During avian embryonic development, maternal thyroid hormones in the egg yolk play an essential role until hatching. In addition, the thyroid gland of the embryo starts to secrete thyroid hormones at around E9 (Darras, 2019). The amount of hormone T_3_ gradually increases in the brain and peaks at E20. In this study, we examined the involvement of thyroid hormones in the imprinting learning process during the pre-hatching period. The administration of MMI did not delay the hatching date; however, we do not deny the possibility that the impairment of imprinting by MMI administration might be caused by the damage to brain function or development, including irreversible defects in motor skills for walking. Since the thyroid hormone at peri-hatch period has been reported to be implicated in the control of muscle development, lung maturation and the switch from chorioallantoic to pulmonary respiration (De Groef et al., 2013), the MMI might impair those developments. Suppose the MMI-administered chicks can be imprinted later than P2 or P3 when the thyroid hormone level fully recovers and the sensitive period is still open. In that case, the data will provide evidence that MMI does not adversely and irreversibly affect brain development and imprinting but causes reversible cognitive deficits. Another concern is that T_3_ levels in the brain usually increase during imprinting training, which might not occur in MMI-administered chicks. We assume that this is unlikely because T_4_ levels in the serum of the MMI-administered chicks already recovered 24 h after hatching, but in such case, the synthesis of thyroid hormones in the thyroid gland may be affected by MMI administration even at P1, and then the chicks will not be imprinted.

The normalized approach distance to the static state of the object in the Training two was significantly correlated with the preference score in the test (Figure 6D). The MMI-administered chicks did not continue the approach behavior toward the static object during Training 2 (Figure 4B). These indicate that the MMI-administered chicks could not establish the approach behavior during Training two and then failed to acquire the preference. There are two possible effects of MMI on the learning process during imprinting. First, MMI may impair innate responsiveness to moving objects. The MMI-administered chicks approached the moving object; however, their approach distances were shorter. In a previous study by Aoki et al. (2022a), strong responsiveness to a moving object was necessary to acquire approach behavior toward an object in a static state. In the study, 12 h after hatching, the chicks approached too weakly to achieve the second learning process, the acquired approach behavior toward the static imprinting object. Thus, the effects on the learning process may have been caused by delays in cognitive development. Second, MMI administration may impair the ability to acquire a strong preference for the imprinting object. Normally, chicks initially acquire a relatively small preference for the imprinting object and then acquire a stronger preference through repeated approaches. However, the MMI-administered chicks seemed to lose a stronger preference for the imprinting object and then habituate it through repeated approaches. These effects on the learning processes will ultimately lead to incomplete memory.

When the Pearson’s product-moment correlation was applied to the data of the PBS-administered chicks, the correlation was not significant between the normalized approach distance in the second period and the preference score, but *r* value was relatively high (0.467). The non-significant correlation may be due to the small sample size of the PBS-administered chicks (*n* = 10), otherwise, the approach behavior toward the static state of imprinting object in the training will not be associated with the preference score in the test.

In conclusion, using a high-time-resolution apparatus based on a running disc, we showed that intrinsic thyroid hormones at the pre-hatching period, just before hatching, play important roles in the learning processes of imprinting as the first experience in the individual life of birds.

## Data Availability

The raw data supporting the conclusion of this article will be made available by the authors, without undue reservation.

## Abbreviations

AIC, Akaike information criterion; GLMM, generalized linear mixed model; MMI, methimazole; T3, triiodothyronine; T4, thyroxine; TMB, 3,3′,5,5′-Tetramethylbenzidine; PBS, phosphate-buffered saline.

## References

[B1] Ahmed R. G. (2015). Hypothyroidism and brain developmental players. Thyroid. Res. 8, 2. 10.1186/s13044-015-0013-7 25878727 PMC4397876

[B2] Akaike M. Kato N. Ohno H. Kobayashi T. (1991). Hyperactivity and spatial maze learning impairment of adult rats with temporary neonatal hypothyroidism. Neurotoxicol Teratol. 13, 317–322. 10.1016/0892-0362(91)90077-a 1886541

[B3] Aoki N. Fujita T. Mori C. Fujita E. Yamaguchi S. Matsushima T. (2020). Blockade of muscarinic acetylcholine receptor by scopolamine impairs the memory formation of filial imprinting in domestic chicks (Gallus *Gallus domesticus*). Behav. Brain Res. 379, 112291. 10.1016/j.bbr.2019.112291 31689441

[B4] Aoki N. Mori C. Fujita T. Serizawa S. Yamaguchi S. Matsushima T. (2022a). Imprintability of newly hatched domestic chicks on an artificial object: A novel high time-resolution apparatus based on a running disc. Front. Physiol. 13, 822638. 10.3389/fphys.2022.822638 35370801 PMC8965712

[B5] Aoki N. Mori C. Fujita T. Serizawa S. Yamaguchi S. Matsushima T. (2022b). Subtype-selective contribution of muscarinic acetylcholine receptors for filial imprinting in newly-hatched domestic chicks. Behav. Brain Res. 424, 113789. 10.1016/j.bbr.2022.113789 35151794

[B6] Aoki N. Yamaguchi S. Fujita T. Mori C. Fujita E. Matsushima T. (2018). GABA-A and GABA-B receptors in filial imprinting linked with opening and closing of the sensitive period in domestic chicks (Gallus gallus domesticus). Front. Physiol. 9, 1837. 10.3389/fphys.2018.01837 30618842 PMC6305906

[B7] Bateson P. P. (1964). Relation between conspicuousness of stimuli and their effectiveness in the imprinting situation. J. Comp. Physiol. Psychol. 58, 407–411. 10.1037/h0045376 14241055

[B8] Bateson P. P. (1966). The characteristics and context of imprinting. Biol. Rev. Camb Philos. Soc. 41, 177–211. 10.1111/j.1469-185x.1966.tb01489.x 5295796

[B9] Bernal J. (2007). Thyroid hormone receptors in brain development and function. Nat. Clin. Pract. Endocrinol. Metab. 3, 249–259. 10.1038/ncpendmet0424 17315033

[B10] Bolhuis J. J. (1991). Mechanisms of avian imprinting: A review. Biol. Rev. Camb Philos. Soc. 66, 303–345. 10.1111/j.1469-185x.1991.tb01145.x 1801945

[B11] Darbra S. Balada F. Marti-Carbonell M. A. Garau A. (2004). Perinatal hypothyroidism effects on step-through passive avoidance task in rats. Physiol. Behav. 82, 497–501. 10.1016/j.physbeh.2004.04.057 15276815

[B12] Darras V. M. (2019). The role of maternal thyroid hormones in avian embryonic development. Front. Endocrinol. (Lausanne) 10, 66. 10.3389/fendo.2019.00066 30800099 PMC6375826

[B13] Darras V. M. Van Herck S. L. Geysens S. Reyns G. E. (2009). Involvement of thyroid hormones in chicken embryonic brain development. Gen. Comp. Endocrinol. 163, 58–62. 10.1016/j.ygcen.2008.11.014 19063893

[B39] De Groef B. Grommen S. V. Darras V. M. (2013). Hatching the cleidoic egg: the role of thyroid hormones. Front Endocrinol (Lausanne) 4, 63 23755041 10.3389/fendo.2013.00063 PMC3668268

[B14] Gilbert M. E. Sui L. (2006). Dose-dependent reductions in spatial learning and synaptic function in the dentate gyrus of adult rats following developmental thyroid hormone insufficiency. Brain Res. 1069, 10–22. 10.1016/j.brainres.2005.10.049 16406011

[B15] Haba G. Nishigori H. Sasaki M. Tohyama K. Kudo K. Matsumura Y. (2014). Altered magnetic resonance images of brain and social behaviors of hatchling, and expression of thyroid hormone receptor βmRNA in cerebellum of embryos after Methimazole administration. Psychopharmacol. Berl. 231, 221–230. 10.1007/s00213-013-3229-z 23949207

[B16] Horn G. (1986). Imprinting, learning, and memory. Behav. Neurosci. 100, 825–832. 10.1037//0735-7044.100.6.825 3545258

[B17] Horn G. (2004). Pathways of the past: The imprint of memory. Nat. Rev. Neurosci. 5, 108–120. 10.1038/nrn1324 14735114

[B18] Horn G. (1998). Visual imprinting and the neural mechanisms of recognition memory. Trends Neurosci. 21, 300–305. 10.1016/s0166-2236(97)01219-8 9683322

[B19] Izawa E. Yanagihara S. Atsumi T. Matsushima T. (2001). The role of basal ganglia in reinforcement learning and imprinting in domestic chicks. Neuroreport 12, 1743–1747. 10.1097/00001756-200106130-00045 11409751

[B20] Jaynes J. (1957). Imprinting: The interaction of learned and innate behavior. II. The critical period. J. Comp. Physiol. Psychol. 50, 6–10. 10.1037/h0044716 13406129

[B21] Kagami K. Nishigori H. Nishigori H. (2010). Effects of prenatal exposure to antithyroid drugs on imprinting behavior in chicks. Physiol. Behav. 101, 297–301. 10.1016/j.physbeh.2010.05.015 20515699

[B22] Lorenz K. Z. (1937). The companion in the bird's world. Auk 54, 245–273. 10.2307/4078077

[B23] Macnabb C. O'hare E. Cleary J. Georgopoulos A. P. (1999). Congenital hypothyroidism impairs response alternation discrimination behavior. Brain Res. 847, 231–239. 10.1016/s0006-8993(99)02038-7 10575092

[B24] Maekawa F. Komine O. Sato K. Kanamatsu T. Uchimura M. Tanaka K. (2006). Imprinting modulates processing of visual information in the visual wulst of chicks. BMC Neurosci. 7, 75. 10.1186/1471-2202-7-75 17101060 PMC1657023

[B25] Matsushima T. Izawa E. Aoki N. Yanagihara S. (2003). The mind through chick eyes: Memory, cognition and anticipation. Zool. Sci. 20, 395–408. 10.2108/zsj.20.395 12719641

[B26] Mccabe B. J. (2013). Imprinting. Wiley Interdiscip. Rev. Cogn. Sci. 4, 375–390. 10.1002/wcs.1231 26304225

[B27] Mccabe B. J. (2019). Visual imprinting in birds: Behavior, models, and neural mechanisms. Front. Physiol. 10, 658. 10.3389/fphys.2019.00658 31231236 PMC6558373

[B28] Mishra I. Bhardwaj S. K. Malik S. Kumar V. (2017). Concurrent hypothalamic gene expression under acute and chronic long days: Implications for initiation and maintenance of photoperiodic response in migratory songbirds. Mol. Cell. Endocrinol. 439, 81–94. 10.1016/j.mce.2016.10.023 27789391

[B29] Mori C. Aoki N. Fujita T. Yamaguchi S. Matsushima T. Homma K. J. (2022). Gene expression profiles of the muscarinic acetylcholine receptors in brain regions relating to filial imprinting of newly-hatched domestic chicks. Behav. Brain Res. 420, 113708. 10.1016/j.bbr.2021.113708 34902480

[B30] Prabhat A. Batra T. Kumar V. (2020). Effects of timed food availability on reproduction and metabolism in zebra finches: Molecular insights into homeostatic adaptation to food-restriction in diurnal vertebrates. Horm. Behav. 125, 104820. 10.1016/j.yhbeh.2020.104820 32710887

[B31] Ramsay A. O. Hess E. H. (1954). A laboratory approach to the study of imprinting. Wilson Bull. 66, 196–206.

[B32] Reyns G. E. Venken K. Morreale De Escobar G. Kuhn E. R. Darras V. M. (2003). Dynamics and regulation of intracellular thyroid hormone concentrations in embryonic chicken liver, kidney, brain, and blood. Gen. Comp. Endocrinol. 134, 80–87. 10.1016/s0016-6480(03)00220-x 13129506

[B33] Shafiee S. M. Vafaei A. A. Rashidy-Pour A. (2016). Effects of maternal hypothyroidism during pregnancy on learning, memory and hippocampal BDNF in rat pups: Beneficial effects of exercise. Neuroscience 329, 151–161. 10.1016/j.neuroscience.2016.04.048 27181637

[B34] Solomonia R. O. Mccabe B. J. (2015). Molecular mechanisms of memory in imprinting. Neurosci. Biobehav Rev. 50, 56–69. 10.1016/j.neubiorev.2014.09.013 25280906 PMC4726915

[B35] Too H. C. Shibata M. Yayota M. Darras V. M. Iwasawa A. (2017). Expression of thyroid hormone regulator genes in the yolk sac membrane of the developing chicken embryo. J. Reprod. Dev. 63, 463–472. 10.1262/jrd.2017-017 28652559 PMC5649095

[B36] Vallortigara G. Versace E. (2018). “Filial imprinting,” in Encyclopedia of animal cognition and behavior. Editors Vonk J. Shackelford T. (cham, Switzerland: Springer).

[B37] Van Herck S. L. Geysens S. Delbaere J. Darras V. M. (2013). Regulators of thyroid hormone availability and action in embryonic chicken brain development. Gen. Comp. Endocrinol. 190, 96–104. 10.1016/j.ygcen.2013.05.003 23707378

[B38] Yamaguchi S. Aoki N. Kitajima T. Iikubo E. Katagiri S. Matsushima T. (2012). Thyroid hormone determines the start of the sensitive period of imprinting and primes later learning. Nat. Commun. 3, 1081. 10.1038/ncomms2088 23011135 PMC3658000

