# Supplementary material for: Temporal hampering of thyroid hormone synthesis just before hatching impeded the filial imprinting in domestic chicks
Source: Front Physiol. 2023 Feb 16;14:1084816. doi: 10.3389/fphys.2023.1084816 (PMC9978523; doi:10.3389/fphys.2023.1084816)
Supplement: Supplementary file 1 [file DataSheet1.PDF]

Supplementary Table 1. *p* values				
			t value	p value
Figure 2	Serum T_4	12 hours before hatching	4.786	0.001
		1 hours after hatching	1.314	0.207
		12 hours after hatching	0.341	0.737
		24 hours after hatching	1.602	0.132
Figure 4A	Training 1	1st period	1.293	0.214
		2nd period	0.472	0.642
		3rd period	-1.183	0.253
		4th period	-1.106	0.283
		5th period	0.322	0.751
Figure 4B	Training 2	1st period	-0.996	0.343
		2nd period	-2.250	0.048
		3rd period	-2.402	0.028
		4th period	-1.633	0.120
		5th period	-0.210	0.837
Figure 6A	Test	Preference score	-2.892	0.014
Figure 6B	Test	Yellow	2.417	0.031
Figure 6C	Test	Blue	0.271	0.790

Supplementary Table 2. Results of *t*-test to estimate differences between the approach distances to the yellow object and those to blue object in the test

Group	ID	approach distances to the yellow object (cm)	approach distances to the blue object (cm)	*p* values
PBS-administration	051922_3	82.89	2.89	0.000
	060922_1	115.11	54.89	0.000
	060922_3	36.98	20.67	0.022
	061622_1	34.36	8.58	0.000
	061622_2	11.60	3.16	0.000
	061622_6	13.96	22.71	0.068
	063022_2	59.02	21.11	0.000
	063022_3	27.29	5.96	0.000
	070722_1	74.31	15.47	0.000
	070722_4	89.16	30.27	0.000
MMI-administration	051922_4	18.98	18.80	0.968
	051922_5	1.91	1.20	0.330
	060922_2	47.47	32.18	0.010
	060922_4	19.38	14.36	0.224
	061622_4	63.24	25.16	0.000
	061622_5	16.13	12.58	0.393
	061622_7	27.11	19.82	0.083
	063022_1	17.42	9.16	0.063
	063022_4	33.91	32.76	0.829
	070722_2	14.04	8.62	0.130
	070722_3	13.78	12.31	0.691

Supplementary note on the generalized linear mixed model (GLMM) analysis on behavioral data in the Training 2 and test.

To examine whether the preference score is associated with approach distances in each period, we constructed generalized linear mixed models (GLMM), and evaluated them using the Akaike information criterion (AIC). We assumed full and null GLM (generalized linear models) as follow. These models had AIC (Akaike Information Criteria) ~ -22.302 (full model) and -20.926 (null model) respectively.

- Full model:

$$Y(Preference\ \ score) = \alpha 0 \ + \ \alpha 1 * \frac{2nd\ \ period}{1st\ \ period} \ + \ \alpha 2 * \frac{3rd\ \ period}{1st\ \ period} \ + \ \alpha 3 * \frac{4th\ \ period}{1st\ \ period} + \alpha 4 * \frac{5th\ \ period}{1st\ \ period}$$

- Null model:

$$Y\ (Preference\ score) = \alpha_0$$

Here, the variables in the formula are defined as:

- $\frac{2nd\ \ period}{1st\ \ period}$ represents the approach distance in the 2nd period normalized by that in the 1st period.
- $\frac{3rd\ \ period}{1st\ \ period}$ represents the approach distance in the 3rd period normalized by that in the 1st period.
- $\frac{4th\ \ period}{1st\ \ period}$ represents the approach distance in the 4th period normalized by that in the 1st period.
- $\frac{5th\ \ period}{1st\ \ period}$ represents the approach distance in the 5th period normalized by that in the 1st period.

Among the all possible combinations, the following a model showed the lowest AIC values [AIC = -27.512]. The other models had higher AIC values and not considered here (Supplementary Table3).

$$Y(Preference\ \ score) = \alpha 0 \ + \ \alpha 1 * \frac{2nd\ \ period}{1st\ \ period}$$

Estimated coefficient α_1 was 0.0683 and the probability that $\alpha_1 > 0$ was lower than $p = 0.05$. From this result, we concluded the approach distance in the 2nd period is more appropriate to acquire the higher preference for the imprinting object rather than other periods.

Supplementary table 3. AIC and log-likelihood in the GLMM

	AIC	Log-likelihood
$Y = \alpha 0$	-20.926	12.463
$Y = \alpha 0 + \alpha 1 * \frac{2nd\ period}{1st\ period}$	-27.512	16.756
$Y = \alpha 0 + \alpha 2 * \frac{3rd\ period}{1st\ period}$	-26.156	16.078
$Y = \alpha 0 + \alpha 3 * \frac{4th\ period}{1st\ period}$	-25.038	15.519
$Y = \alpha 0 + \alpha 4 * \frac{5th\ period}{1st\ period}$	-21.737	13.868
$Y = \alpha 0 + \alpha 1 * \frac{2nd\ period}{1st\ period} + \alpha 2 * \frac{3rd\ period}{1st\ period}$	-26.282	17.141
$Y = \alpha 0 + \alpha 1 * \frac{2nd\ period}{1st\ period} + \alpha 3 * \frac{4th\ period}{1st\ period}$	-26.285	17.142
$Y = \alpha 0 + \alpha 1 * \frac{2nd\ period}{1st\ period} + \alpha 4 * \frac{5th\ period}{1st\ period}$	-25.704	16.852
$Y = \alpha 0 + \alpha 2 * \frac{3rd\ period}{1st\ period} + \alpha 3 * \frac{4th\ period}{1st\ period}$	-24.867	16.434
$Y = \alpha 0 + \alpha 2 * \frac{3rd\ period}{1st\ period} + \alpha 4 * \frac{5th\ period}{1st\ period}$	-24.317	16.158
$Y = \alpha 0 + \alpha 3 * \frac{4th\ period}{1st\ period} + \alpha 4 * \frac{5th\ period}{1st\ period}$	-23.112	15.556
$Y = \alpha 0 + \alpha 1 * \frac{2nd\ period}{1st\ period} + \alpha 2 * \frac{3rd\ period}{1st\ period} + \alpha 3 * \frac{4th\ period}{1st\ period}$	-24.299	17.15
$Y = \alpha 0 + \alpha 1 * \frac{2nd\ period}{1st\ period} + \alpha 2 * \frac{3rd\ period}{1st\ period} + \alpha 4 * \frac{5th\ period}{1st\ period}$	-24.284	17.142
$Y = \alpha 0 + \alpha 1 * \frac{2nd\ period}{1st\ period} + \alpha 3 * \frac{4th\ period}{1st\ period} + \alpha 4 * \frac{5th\ period}{1st\ period}$	-24.286	17.143
$Y = \alpha 0 + \alpha 2 * \frac{3rd\ period}{1st\ period} + \alpha 3 * \frac{4th\ period}{1st\ period}) + \alpha 4 * \frac{5th\ period}{1st\ period}$	-22.964	16.482
$Y = \alpha 0 + \alpha 1 * \frac{2nd\ period}{1st\ period} + \alpha 2 * \frac{3rd\ period}{1st\ period} + \alpha 3 * \frac{4th\ period}{1st\ period} + \alpha 4 * \frac{5th\ period}{1st\ period}$	-22.302	17.151
